# Methodological Challenges in Determining Longitudinal Associations Between Anticholinergic Drug Use and Incident Cognitive Decline

**DOI:** 10.1111/jgs.12632

**Published:** 2014-01-13

**Authors:** Mandavi Kashyap, Sylvie Belleville, Benoit H Mulsant, Sarah N Hilmer, Amelie Paquette, Le Mai Tu, Cara Tannenbaum

**Affiliations:** * Centre de Recherche, Institut Universitaire de Gériatrie de Montréal, Université de MontréalMontreal, Quebec, Canada; † Department of Psychiatry, Centre for Addiction and Mental Health, University of TorontoToronto, Ontario, Canada; ‡ University of Sydney, Royal North Shore Hospital and Kolling Institute of Medical ResearchSt Leonards, New South Wales, Australia; § Institut Universitaire de Gériatrie de Sherbrooke, Université de SherbrookeSherbrooke, Quebec, Canada

**Keywords:** anticholinergic drug exposure, cognitive decline, longitudinal study, older adults, methods

## Abstract

**Objectives:**

To compare the effect of using different anticholinergic drug scales and different models of cognitive decline in longitudinal studies.

**Design:**

Longitudinal cohort study.

**Setting:**

Outpatient clinics, Quebec, Canada.

**Participants:**

Individuals aged 60 and older without dementia or depression (n = 102).

**Measurements:**

Using baseline and 1-year follow-up data, four measures of anticholinergic burden (anticholinergic component of the Drug Burden Index (DBI-Ach), Anticholinergic Cognitive Burden (ACB), Anticholinergic Drug Scale (ADS), and Anticholinergic Risk Scale (ARS)) were applied. Three models of cognitive decline (worsening of raw neuropsychological test scores, Reliable Change Index (RCI), and a standardized regression based measure (SRB)) were compared in relation to *Diagnostic and Statistical Manual of Mental Disorders, Fifth Edition* (DSM-V) criteria for the onset of a new mild neurocognitive disorder. The consistency of associations was examined using logistic regression.

**Results:**

The frequency of identifying individuals with an increase in anticholinergic burden over 1 year varied from 18% with the DBI-Ach to 23% with the ACB. The frequency of identifying cognitive decline ranged from 8% to 86% using different models. The raw change score had the highest sensitivity (0.91), and the RCI the highest specificity (0.93) against DSM-V criteria. Memory decline using the SRB method was associated with an increase in ACB (odds ratio (OR) = 5.3, 95% confidence interval (CI) = 1.1–25.8), ADS (OR = 5.7, 95% CI = 1.1–27.7), and ARS (OR = 6.5, 95% CI = 1.34–32.3). An increase in the DBI-Ach was associated with a decline on memory testing using the raw change score method (OR = 4.2, 95% CI = 1.8–15.4) and on the Trail-Making Test Part B using SRB (OR = 2.9, 95% CI = 1.1–8.0). No associations were observed using the DSM-V criteria or RCI method.

**Conclusion:**

The choice of different methods for defining drug exposure and cognitive decline will have a significant effect on the results of pharmacoepidemiological studies.

The loss of cholinergic neurons observed in individuals with Alzheimer's disease provides a theoretical rationale why exposure to anticholinergic drugs may contribute to cognitive impairment,^1^ yet only a fraction of clinical trials testing individual anticholinergic agents have systematically measured and reported cognitive outcomes.^2^ In the few trials that have done so, severe cognitive deficits rarely become apparent.^2^,^3^ Inconsistent results from longitudinal studies on the relationship between cumulative anticholinergic exposure and cognitive decline lend further uncertainty to the question of whether anticholinergic drugs can be safely prescribed on a chronic basis.^4^–^7^

One challenge relates to the fact that the findings from these studies are difficult to interpret and compare. Different methods are often used for measuring changes in anticholinergic drug exposure and incident cognitive decline.^4^–^11^ Several anticholinergic drug scales exist, including the anticholinergic component of the Drug Burden Index (DBI-Ach),^8^ the Anticholinergic Cognitive Burden Scale (ACB),^11^ the Anticholinergic Drug Scale (ADS),^10^ and the Anticholinergic Risk Scale (ARS).^9^ Derivation of the scores from each method differs. The DBI-Ach is established from drugs with clinical anticholinergic effects with exposure quantified using extrapolation of pharmacological principles of cumulative drug exposure and dose-response. The other three measures rely to a variable extent on each drug's serum anticholinergic activity according to in vitro assay and demonstrable clinical effects.

Different models also exist for measuring cognitive decline using neuropsychological test batteries with or without clinical assessment. A negative change in raw test score performance, using the absolute unstandardized difference between baseline and retest scores, is the simplest way of assessing whether performance has worsened. Cognitive decline has been categorized as the lowest quintile of the difference between baseline score and follow-up visit,^5^ but the confounding effects of regression to the mean or practice effects may influence raw change scores.^12^ A reliable and clinically meaningful change in cognition should ideally be defined as one that exceeds fluctuations in test scores secondary to measurement error and other factors known to influence serial test results. The reliable change index (RCI) and the standardized regression-based measure (SRB) are two methods that attempt to take these factors into consideration.^13^–^16^ The RCI provides a confidence interval (CI) for predicted change by taking into account the test–retest reliability of the measure.^13^,^16^ The SRB uses a more-sophisticated statistical approach that enables adjustment for practice effects, regression to the mean, and age-associated normal cognitive decline.^14^–^16^ In turn, a clinically relevant change can be defined by progression from a normal cognitive state to meeting *Diagnostic and Statistical Manual of Mental Disorders, Fifth Edition* (DSM-V) criteria for a mild neurocognitive disorder (previously referred to as mild cognitive impairment).^17^ It stands to reason that the results of each of these analyses will produce diverse estimates of cognitive decline.

The aim of this study was to explore the extent to which an interrelationship between different anticholinergic drug scales and different models for measuring cognitive decline can affect the results of longitudinal studies. Using data from the same cohort of older adults followed over a 1-year period, this analysis compares changes in anticholinergic drug scales, estimates the frequency with which different models identify individuals with cognitive decline, compares the sensitivity and specificity of these cognitive models with those of the new DSM-V criteria for mild neurocognitive disorder, and determines the effect on estimates of associations between anticholinergic drug exposure and cognitive decline. A brief discussion will follow to address the question of which—if any—definition of cognitive change might be most appropriate.

## Methods

### Study Setting, Design, and Participants

Data for this analysis were derived from a longitudinal cohort study with repeated neuropsychological test measures performed at baseline and 1-year follow-up. Cohort participants were French-speaking adult men and women aged 60 and older recruited from outpatient incontinence clinics in the Montreal and Sherbrooke areas of Quebec, Canada. Criteria that excluded individuals from entering the cohort were the presence of frank cognitive impairment (Mini-Mental State Examination (MMSE) score ≤24)^18^ or clinical depression (Geriatric Depression Scale score >11)^19^ at baseline. Members of the cohort without missing neuropsychological test data were included in this analysis. The ethics review boards of the Montreal and Sherbrooke study sites approved the study, and informed consent was obtained from each participant before data collection.

### Different Measures of Anticholinergic Drug Exposure

Participants self-reported medication use during baseline and 1-year follow-up interviews with a research nurse. Self-report was validated against medication boxes, and prescription refills were checked with the pharmacist when needed to ascertain adherence. Each medication and its frequency of administration and dose were recorded. Cumulative anticholinergic drug exposure for each participant was quantified using the DBI-Ach,^8^ ACB,^11^ ADS,^10^ and ARS.^9^ For the DBI-Ach the formula

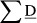


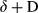
was used to calculate anticholinergic drug burden, with D the daily dose of each drug with anticholinergic properties taken by the participant and δ the minimum effective daily dose recommended in the Compendium of Pharmaceuticals and Specialities from the Canadian Pharmacists Association.^20^ The minimum recommended daily dose is used as an estimate of the dose required to achieve 50% of the maximum anticholinergic activity (DR50). To determine ACB, ADS, and ARS scores, participants' medications were assigned points according to the published lists for each scale and summed together for a total score. With all four instruments, higher scores indicate higher anticholinergic burden. Each scale can yield a different anticholinergic burden score for the same individual profile because the criteria for determining anticholinergic effects differ with respect to specific drugs and drug classes and the type of evidence considered in the development of the three measures.

### Models of Cognitive Decline

A standardized battery of global, memory, and nonmemory neuropsychological tests was administered to each participant at baseline and 1-year follow-up. Global cognitive status was assessed using the Mattis Dementia Rating Scale, MMSE, and Montreal Cognitive Assessment (MoCA);^12^ memory using Free and Cued Recall (RL/RI) and the Rey-Osterrieth Complex Figure Test recall;^12^ and nonmemory using the Digit Symbol-Coding subtest of the Wechsler Adult Intelligence Scale, the Trail-Making Test, and the Stroop test (Victoria version).^12^ Participants also reported subjective memory complaints, measured using the Questionnaire d'auto-évaluation de la mémoire,^21^ and completed the Functional Autonomy Measurement Systems questionnaire.^22^

The occurrence of cognitive decline was assessed in three different ways for each participant: worsening in the raw neuropsychological test scores for each test; a change in scores on each test that exceeded an expected cutoff based on the test–retest RCI;^13^,^16^ and a change in scores on each test that persisted when regression to the mean, practice effects, and age were accounted for using the SRB method.^14^–^16^ Data from an additional 29 participants with MMSE scores of 27 or greater, MoCA scores of 26 or greater, and absence of use of any anticholinergic or sedative drugs at the baseline and 1-year follow up visits were used for calculation of expected change for the RCI and SRB-based methods.

### DSM-V Criteria for a Mild Neurocognitive Disorder

A geriatrician (CT) and an experienced neuropsychologist (SB), blinded to each participant's drug exposure status, independently classified participants as having normal cognition or meeting DSM-V criteria for a mild neurocognitive disorder at baseline and again at 1-year follow-up. To meet DSM-V criteria for a mild neurocognitive disorder, participant data had to include a subjective cognitive complaint, evidence of an objective cognitive deficit on neuropsychological tests defined as at least one test score with a *z*-score smaller than −1 or a score less than 26 on the MoCA, no indication of significant functional impairment on the Functional Autonomy Measurement Systems questionnaire or according to the research assistant's judgment, and no evidence of delirium or clinical depression (Geriatric Depression Scale score <12).^17^ To establish whether participants had a subjective cognitive complaint, the evaluators agreed that participants would be coded positively if they had a mean score greater than 2 for difficulty remembering conversations or a mean score greater than 2.5 for difficulty remembering movie elements on the Questionnaire d'auto-évaluation de la mémoire, based on a previous validation study in individuals with mild cognitive impairment.^23^

### Statistical Analysis

Descriptive statistics were used to quantify and compare changes in the different anticholinergic drug scales and the different cognitive measures at baseline and 1-year follow-up. An increase in anticholinergic burden corresponded to positive values resulting from subtraction of each baseline from its 1-year follow-up anticholinergic score. Cognitive change in relation to the new DSM-V criteria for mild neurocognitive impairment was expressed as the sensitivity and specificity of detecting cognitive decline on any one of the neuropsychological tests using the raw change, RCI, and SRB methods. Potential associations between an increase in anticholinergic burden and incident cognitive impairment (binary outcome) were explored using univariate logistic regression for each of the different definitions, reported as odds ratios (ORs) with 95% CIs. An increase in anticholinergic burden was treated continuously and categorically, but only results from binary analyses are reported. The number needed to harm was calculated as the inverse of the absolute difference in event rates of new cognitive decline between the exposed and nonexposed groups.^24^

## Results

One hundred two members of the observational cohort met inclusion criteria. The mean age ± SD of these participants was 71.9 ± 7.3, participants reported on average 12.5 ± 3.7 years of education, and 84% were female. The mean number of comorbid conditions was 6 ± 3. The mean number of prescription medications at baseline was 7 ± 4.

### Comparing Changes in Anticholinergic Drug Scales

Table1 shows the magnitude of change for each drug scale and the number of participants whose anticholinergic burden increased over the 1-year period. The DBI-Ach identified the fewest number of individuals with an increase in anticholinergic burden (n = 18), and the ACB identified the most (n = 23). A frequency list of the anticholinergic drugs added to the participants' medication regimen over the 1-year period included solifenacin (n = 8), trospium chloride (n = 5), oxybutynin (n = 3), tolterodine (n = 2), trazodone (n = 4), paroxetine (n = 2), levodopa (n = 2), sertraline (n = 1), clonazepam (n = 1), and lorazepam (n = 1). Differences in change scores were attributable to inclusion of paroxetine in the ACB (score of 3) and DBI-Ach only, inclusion of trazodone (score of 1) in the ACB only, inclusion of levodopa (score of 1) in the ARS only, and the remaining nonantimuscarinic drugs exclusively in the ADS (score of 1 each). All antimuscarinic agents received a score of 3 on the ADS, ARS, and ACB except for tolterodine, which was attributed a value of 2 on the ARS scale.

**Table 1:** Comparison of 1-Year Changes on the Anticholinergic Drug Scales

Scale	1-Year Incidence of an Increase in Anticholinergic Burden, n (%)	Baseline Score	1-Year Score	Magnitude of Change in Score
		Mean ± SD (Range)		
Anticholinergic component of the drug burden index	18 (17.6)	0.1 ± 0.2 (0–1)	0.6 ± 0.2 (0–1)	0.5 ± 0.2 (0.0–0.7)
Anticholinergic risk scale	20 (19.6)	0.2 ± 0.5 (0–2)	3.1 ± 0.7 (2–5)	2.9 ± 0.4 (2–3)
Anticholinergic drug scale	22 (21.6)	0.4 ± 0.5 (0–1)	3.1 ± 1.1 (1–5)	2.7 ± 0.8 (1–4)
Anticholinergic cognitive burden scale	23 (22.5)	0.6 ± 1.0 (0–4)	3.4 ± 1.0 (2–7)	2.8 ± 0.5 (2–4)

### Differences in Models of Cognitive Decline

The 1-year rates of cognitive decline that the raw change score, RCI, and SRB methods detected are shown in Table2 according to the different cognitive tests that participants performed. When cognitive decline is defined as worsening on at least one of these tests, the RCI method identified cognitive decline in eight participants, the raw change method in 88, and the SRB method in 66.

**Table 2:** Cognitive Decline According to Various Tests in Older Adults (n = 102)

Test	Raw Change Method		Reliable Change Index Method		Standardized Regression-Based Method	
	1-Year Incidence, n (%)	Mean Change ± SD (Range)	1-Year Incidence, n (%)	Mean Change ± SD (Range)	1-Year Incidence, n (%)	Mean Change ± SD (Range)
Mini-mental state examination	24 (23.5)	0.2 ± 0.4 (0–1)	2 (2.0)	0.3 ± 0.9 (−3.2–2.7)	18 (17.6)	−0.1 ± 1.6 (−6.6–3.0)
Mattis dementia rating scale	31 (30.4)	0.3 ± 0.5 (0–1)	1 (0.9)	0.2 ± 0.7 (−1.8–3.9)	26 (25.5)	−0.9 ± 1.7 (−7.2–1.9)
Memory neuropsychological tests						
Free and cued recall delayed memory test	12 (11.7)	0.1 ± 0.3 (0–1)	0 (0.0)	0.3 ± 0.9 (−1.6–3.5)	7 (6.9)	1.1 ± 1.9 (−4.9–5.4)
Rey figure—delayed	37 (36.3)	0.4 ± 0.5 (0–1)	2 (2.0)	0.2 ± 0.9 (−2.3–3.2)	5 (4.9)	0.5 ± 0.7 (−2.2–1.4)
Nonmemory tests						
Wechsler Adult Intelligence Scale CODING	33 (32.3)	0.3 ± 0.5 (0–1)	2 (2.0)	0.2 ± 1.0 (−4.5–2.6)	10 (9.8)	−0.6 ± 0.9 (−4.7–1.4)
Trail-Making Test Part B	41 (40.2)	0.4 ± 0.5 (0–1)	1 (0.9)	−0.1 ± 0.9 (−5.1–4.4)	41 (40.2)	1.5 ± 2.3 (−2.5–13.0)
Stroop color time	29 (28.4)	0.3 ± 0.5 (0–1)	0 (0.0)	−0.2 ± 0.8 (−5.2–1.6)	28 (27.5)	0.8 ± 1.8 (−4.9–7.9)
At least one neuropsychological test	88 (86.3)	—	8 (7.8)	—	66 (64.7)	—

SD = Standard Deviation.

### Cognitive Change in Relation to the New DSM-V Criteria for Mild Neurocognitive Impairment

**Figure 1:**
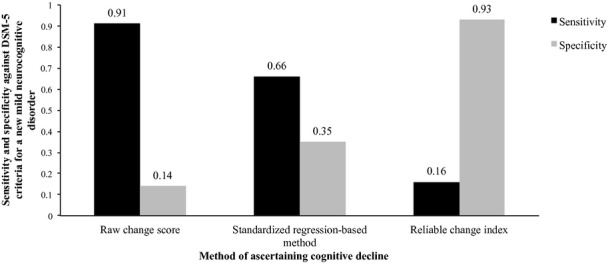
Sensitivity and specificity of different methods of measuring cognitive decline (worsening performance on at least one neuropsychological test) against *Diagnostic and Statistical Manual of Mental Disorders, Fifth Edition* (DSM-V), diagnosis of mild neurocognitive impairment at 1-year follow-up.

Twelve participants with previously normal cognition progressed to a DSM-V diagnosis of a new mild neurocognitive disorder over the 1-year period. Figure1 shows the sensitivity and specificity of each cognitive change method for identifying individuals with a new mild neurocognitive disorder. The raw change score method had the highest sensitivity (91%), the RCI had the highest specificity (93%), and the SRB method showed a compromise between the two.

### Effect of the Different Anticholinergic Scales and Cognitive Change Methods on Associations Between Anticholinergic Drugs and Cognitive Decline

No significant associations were found in analyses that regressed an increase in the anticholinergic drug scales on the occurrence of cognitive decline according to DSM-V criteria or the RCI method. Using the SRB method, a positive relationship was found between an increase in anticholinergic burden on the DBI-Ach and poorer performance on the Trail-Making Test Part B test (OR = 2.2, 95% CI = 1.1–8.06) and between the ACB (OR = 5.3, 95% CI = 1.1–25.8), ADS (OR = 5.7 95% CI = 1.1–27.7), or ARS (OR = 6.5, 95% CI = 1.3–32) and poorer RL/RI delayed memory. The only significant relationship that the raw change score method revealed was between an increase on the DBI-Ach and poorer RL/RI delayed memory performance (OR = 4.2, 95% CI = 1.8–15.4).

The number of participants with an increase in anticholinergic drug scale required to cause one incident case of cognitive decline (number needed to harm) can only be calculated when significant relationships are found. Therefore, no number needed to harm could be calculated for the development of a new DSM-V diagnosis of a mild neurocognitive disorder. To illustrate an example of differences in the number-needed-to-harm statistic for worsening on the RL/RI delayed memory test using the SRB method, Table3 shows the effect of different anticholinergic drug scales on the calculation of the number-needed-to-harm statistic.

**Table 3:** Example of the Effect of Different Anticholinergic Scales on Associations with Cognitive Decline Using Changes in Performance on the Delayed Memory Test According to the Standardized Regression-Based Method

Anticholinergic Drug Scale	Decline on the Free and Cued Recall—Delayed Memory Test, Odds Ratio (95% Confidence Interval)	Number Needed to Harm^a^
Increase on the anticholinergic component of the drug burden index	4.0 (0.8–19.7)	—
Increase on the anticholinergic cognitive burden scale	5.3 (1.1–25.8)	8
Increase on the anticholinergic drug scale	5.7 (1.1–27.7)	7
Increase on the anticholinergic risk scale	6.5 (1.3–32.3)	6

a Number needed to harm is the inverse of the absolute difference in the event rates for cognitive decline between the exposed and nonexposed groups.

## Discussion

This analysis shows that the method chosen to measure and calculate changes in anticholinergic drug exposure and incident cognitive decline can have a significant effect on the results of causal association studies. Changes in anticholinergic scores differed for the same individuals depending on the anticholinergic scale applied. The incidence of cognitive decline also exhibited a wide range of values, from 8% to 86%. Using DSM-V criteria as the criterion standard, the raw change score was most sensitive and the RCI most specific. No association was found between an increase in anticholinergic burden and the diagnosis of a new mild neurocognitive disorder, although other methods of measuring cognitive decline variably detected associations. For example, six, seven, and eight individuals would need to increase their anticholinergic burden on the ARS, ADS, and ACB scales, respectively, to detect one additional case of worsening memory performance using the SRB method.

Results from previous longitudinal studies examining the effect of anticholinergic drug use on cognitive decline can be interpreted in light of these findings. The raw change score detects cognitive deficits at a low threshold, including many individuals who do not meet DSM-V criteria. Studies that used the raw change score method found positive associations between higher anticholinergic drug scores and incident cognitive decline. For example, the Medical Research Council Cognitive Function and Aging study measured anticholinergic load using the ACB and found that baseline use of anticholinergic drugs was associated with a 0.33-point greater decline (95% CI = 0.03–0.64) in MMSE score over 2 years,^7^ whereas studies that used a clinical definition of cognitive change were less likely to find associations.^6^ The categorization of anticholinergic drug load also played a role; a 6-year longitudinal study of African Americans found variations in the risk of developing incident clinically defined cognitive impairment depending on how drug exposure was defined.^6^ Different methods of defining cumulative or intermittent exposure, as well as the number of drugs with possible or definite anticholinergic activity, significantly affected the results of adjusted regression analyses.

No consensus exists on the appropriate definition of true or meaningful cognitive decline. To clinicians, the DSM-V criteria will likely hold the greatest face validity because they include subjective and objective cognitive deficits and leave room for clinical judgment. Methods such as the raw change score do not take into account normal test–retest variability and will detect cognitive deficits at a level that does not meet clinical diagnostic criteria. For this reason, they are more sensitive to false positives. Clinicians may be unwilling to accept such a high rate of false positives in their practice. Researchers, on the other hand, may appreciate the merits of a highly sensitive over a highly specific method depending on its intended use. For example, a highly sensitive method may be the measure of choice for investigating new performance decrements during preclinical screening for early progression to dementia.

This methodological analysis was exploratory in nature and is underpowered to draw conclusions about harmful relationships between the use of anticholinergic drugs and cognitive decline. The goal was to show that failure to take into account practice effects and regression to the mean of neuropsychological test scores in large population-based samples risks misclassifying individuals as false positives—if DSM-V criteria are used as the criterion standard—thereby overestimating associations where none truly exist.^25^ Limitations include the possibility that a temporary increase in anticholinergic burden between interviews could not be accounted for. Studies of single-dose anticholinergics and case reports of long-term anticholinergic therapy suggest that inducible changes in cognition are reversible,^26^ but the time frame for reversibility is likely to depend on pharmacokinetics (clearance) and pharmacodynamics (dissociation from muscarinic receptors in the brain) and is not well described for the wide range of anticholinergic medicines and older population in this study. Some participants had a decrease in their anticholinergic exposure from baseline, but these were grouped with the stable participants in the number-needed-to-harm calculations. Antimuscarinic agents that were not listed on the anticholinergic drug scales were assumed to have a score of 3, which requires validation.

The methods used to measure change in anticholinergic burden and cognitive decline will have a significant effect on the results of causal association studies. Researchers should be aware of these differences and select study instruments and analytical methods that best fit the goals of their study. Conducting a sensitivity analysis using two or more methods may be one way to improve confidence in the conclusions.
